# Ruthenium complex based nanocomposite film with enhanced and selective electrochemical sensing of bifenthrin pesticide†

**DOI:** 10.1039/d4ra04188g

**Published:** 2024-09-18

**Authors:** Sanjeev Bhandari, Bhaskar Sen, Snehadrinarayan Khatua, L. Robindro Singh, Vijay Singh Parihar, Mrityunjoy Mahato

**Affiliations:** a Physics Division, Department of Basic Sciences and Social Sciences, School of Technology, North-Eastern Hill University Shillong Meghalaya 793022 India mrityunjoyedu@gmail.com; b Centre for Advanced Studies, Department of Chemistry, North-Eastern Hill University Shillong Meghalaya 793022 India; c Department of Nanotechnology, School of Technology, North-Eastern Hill University Shillong Meghalaya 793022 India; d Biomaterials and Tissue Engineering Group, Faculty of Medicine and Health Technology, Tampere University 33720 Tampere Finland vijay.parihar@tuni.fi

## Abstract

Bifenthrin (BF), a widely used pyrethroid pesticide in farming, lacks highly sensitive and selective sensors despite its extensive application. Ruthenium complexes are very effective for selective sensing applications but suffer from structural instability at elevated conditions, electrochemical activity, and the use of costly electrolytes. This work improves their electrochemical activity and mechanical strength by incorporating silver nanowires and replacing the costly electrolyte with abundant KCl + PBS, resulting in enhanced signal performance. Herein, a ruthenium complex containing composite film was immobilized on a platinum (Pt) electrode using Langmuir Blodgett technique. The fabricated sensor has been characterized by differential pulse voltammetry (DPV) based electrochemical technique. The BF pesticide sensing parameters, including the limit of detection (LOD), linear range (LR), and sensitivity, were evaluated using SWV, DPV, and CV techniques. Among these, the DPV technique demonstrated the best performance, achieving a sensitivity of 0.648 μA cm^−2^ μM^−1^, a LR of 1–10 μM, and a LOD of 1 μM. The relative standard deviation (RSD) values using DPV are found to be 6.3% (repeatability study), 3% (reproducibility study), 8% (metal ion interference), 5% (organic species interference), and 2% (real sample study), which are much lesser than the World Health Organization (WHO) recommendation of RSD value on the pesticide (*i.e.* 20%). The BF sensor demonstrated a selectivity of 2× difference of peak height response compared to similar pesticides. The reported pesticide sensor will open new options for sensor research using metal complex-based LB film nanocomposite.

## Abstract 2

Bifenthrin (BF), a widely used pyrethroid pesticide in farming, lacks highly sensitive and selective sensors despite its extensive application.

## Graphical abstract

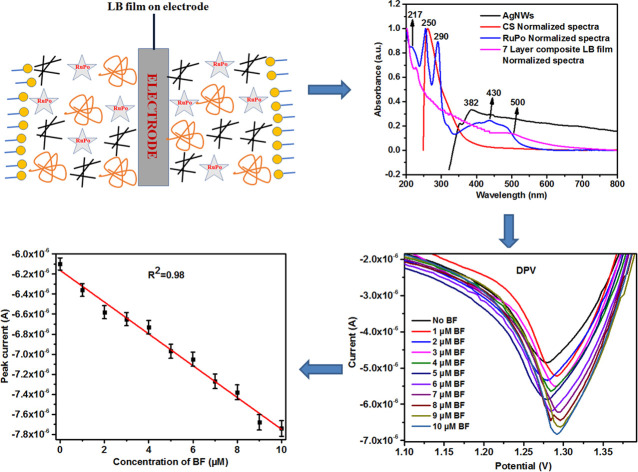

## 1. Introduction

The development of sensitive, selective, and portable pesticide sensors is of growing importance in agro farms as well as food and environment monitoring.^1^ The consumption of pesticides has increased since 1949 in agriculture, veterinary care, and medicine.^2^ As per 2021 data, the global consumption of pesticides is 2.66 million metric tons (MMT).^3–5^ Pesticides are non-degradable toxic chemicals harming the soil and ecology^6^ and long-term consumption of crops treated with pesticides can lead to diseases such as kidney dysfunction, asthma, leukemia, *etc.*^7^

Bifenthrin (BF) belongs to the pyrethroid group of pesticides derived from pyrethrins,^8,9^ which is insoluble in water (solubility of 0.1 mg L^−1^) and has a half-life of 7 days to 8 months in soil.^10^ BF is effective against vegetable pests,^11^ fruit pests,^12^ and mosquitoes.^13^ The acceptable daily intake (ADI) of BF is 0.02 mg kg^−1^,^10^ and any excess can lead to health-related problems such as hepatotoxic, immunotoxic, and neurotoxicity.^14,15^ Hence, there is a need for sensitive and selective methods for the detection of BF pesticides in food samples, water, and soil at very low concentrations. However, there exist conventional equipment/techniques to analyze pesticides, such as high-performance liquid chromatography (HPLC) and gas chromatography-mass spectrometry (GC-MS), which are costly, not portable, time-consuming, require sample preparation, sample pretreatment, skilled manpower, and sophisticated instruments.^16–18^ Developing nanotechnology-based enzymatic biosensors, colorimetric optical sensors, and electrochemical sensors represents a focused effort to address the growing demand for BF pesticide-sensing.^19–25^ In this regard, electrochemistry-based techniques, such as cyclic voltammetry (CV), square wave voltammetry (SWV), and differential pulse voltammetry (DPV) provide highly sensitive sensors to meet the conventional challenges for pesticide detection.^2,26–28^ Nanotechnology and electrochemistry-based sensors can provide highly sensitive sensors. However, it is lacking on other sensor parameters, such as selectivity, reproducibility, and repeatability.

In this context, synthetic metal–organic complexes (MOC), specifically ruthenium(ii) polypyridine (RuPo) complexes, are reported to be active compounds with high selectivity and reproducibility promise for sensor design by optical and electrochemical methods.^29–31^ However, the chemical structure of a pure RuPo complex is not very stable at elevated conditions of temperature, acidic and alkaline conditions. Also, the electrochemical redox activity of RuPo demands costly electrolytes like tetra-*n*-butylammonium perchlorate (TBAP), and its electrocatalytic activity is poor in aqueous solvent, which restricts the practical realization of RuPo compound for sensor design.

In this regard, there are research trends to improve the functionality and structural stability of RuPo by covalent chemical modification and non-covalent complexation with nanomaterials, organic compounds, or polymers. Malins *et al.* reported rhodamine dye doped RuPo complex and immobilized it to glass substrate by sol–gel and dip-coating technique for optical pH sensor.^32^ Xu *et al.* modified the RuPo complex by thioether groups and achieved better selectivity of mercury ions using UV-vis absorption spectra.^33^ Jose *et al.* linked RuPo complexes by catechol derivative for higher fluoride interaction and sensing.^34^ Ramachandran *et al.* added calix[4]arene moiety to RuPo complexes for higher selectivity to Cu and sulfide ions sensing using emission spectra.^35^ There are reports on the increase of electrocatalytic activity of RuPo by incorporating it in LB films of RuPo-stearic acid, RuPo-PANI complex for taste sensors, dopamine sensors, *etc.*^36,37^ Chun *et al.* covalently attached the RuPo complex on a glass substrate and showed an increase in its structural stability and demonstrated an oxygen sensor with higher sensitivity using emission spectroscopy.^38^ Wahl *et al.* reported an increase in the electroactivity of the RuPo compound in complex with stearic acid by LB technique.^39^ Cesca *et al.* reported the impact of the redox properties of RuPo when it was complexed with oxadiazole-based C-chain and immobilized onto LB film.^40^ The present RuPo complexes have been reported by our group for sensing dichlorvos pesticides in solution using emission spectroscopic technique.^41^ Also, our group previously reported another amphiphilic organometalic cobalt complex with varying alkyl chain length and studied their Langmuir monolayer surface properties.^42^ There are several reports on chemical modification of RuPo compounds that improve the functionality of sensors like sensitivity and selectivity. However, rare reports focused on improving the structural stability of RuPo for sensor design. We propose a hypothesis that the structural stability of RuPo may be enhanced by organizing it on a solid support using the 2D LB film deposition method with control orientation, structure, and thickness, as well as using high mechanical strength AgNWs filler and chitosan polymer matrix support in line with earlier report.^30,43–48^

Herein, we report on the electrochemical sensing of BF pesticide using RuPo as a MOC-based active compound incorporated into chitosan and AgNWs-based nanocomposite LB film. The nanocomposite LB film consisted of RuPo complex, octadecylamine (ODA) amphiphile, PVP-AgNWs, and chitosan immobilized on a platinum (Pt) electrode. The PVP-linked AgNWs with high electrical conductivity ∼5840.4 S cm^−1^,^49^ high mechanical property (tensile strength) ∼1.15 GPa,^50^ and high surface area, have been incorporated into RuPo LB film to improve the electrocatalytic performance of the sensor as well as the mechanical strength of the composite LB film. The ODA amphiphile has been used to make a floating composite on the water subphase. The CS + AgNWs + RuPo + ODA Langmuir film shows higher compressibility than Langmuir film in the absence of AgNWs (CS + RuPo + ODA). The LB composite film has been successfully transferred to Pt electrodes and used for sensing BF pesticide. The reported BF pesticide sensor achieved sensing parameters such as limit of detection (LOD), linear range (LR), and sensitivity using SWV, DPV, and CV techniques. The DPV technique showed better sensing performance than CV and SWV technique, such as sensitivity (0.648 μA cm^−2^ μM^−1^), LR (1–10 μM), and LOD (1 μM). The RSD values using DPV, were found to be 6.3% (repeatability study), 3% (reproducibility study), 8% (metal ion interference), 5% (organic species interference), and 2% (real sample study), which are much lesser than the limit of WHO (*i.e.* 20%). The developed sensor demonstrated BF selectivity with 2× difference in peak (height) current response compared to other similar pesticides. The reported pesticide sensor will open new options for sensor research using RuPo and other MOC based LB film active sensor platforms.

## 2. Materials and methods

### 2.1 Chemicals and reagents

**Fig. 1:**
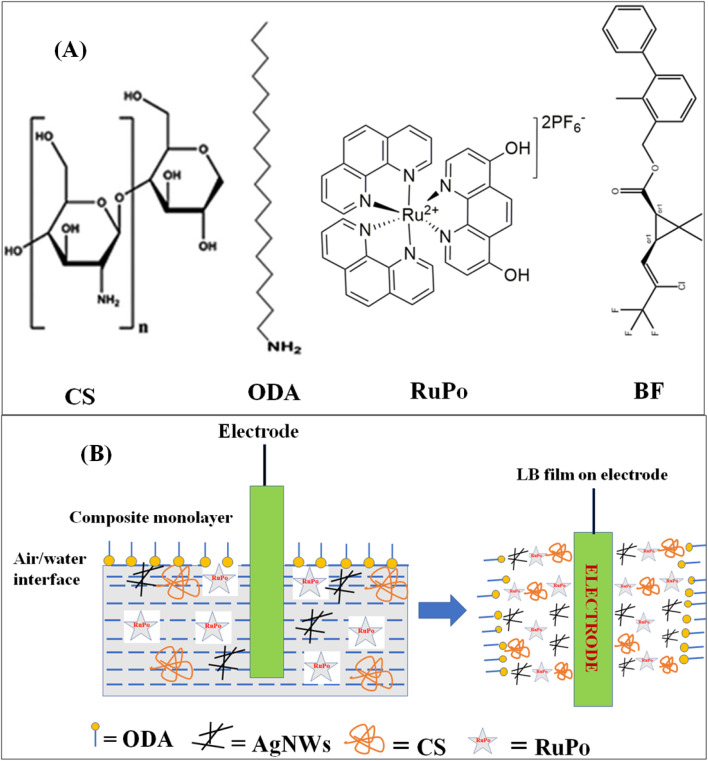
(A) Molecular structure of chitosan (CS), octadecylamine (ODA), ruthenium polypyridine complex (RuPo), and bifenthrin (BF) used in the present work, (B) schematic representation of the fabrication of composite LB film.

All pesticides such as bifenthrin (PESTANAL, Analytical Standard), tetramethrin (PESTANAL, Analytical Standard), fenpropathrin (PESTANAL, Analytical Standard), cyfluthrin (PESTANAL, Analytical Standard), deltamethrin (PESTANAL, Analytical Standard), octadecylamine (ODA, 99%), stearic acid (SA, 98%), arachidic acid (AA, 99%), potassium nitrate (99%), acetonitrile (99.9%), *cis*-bis(2,2′-bipyridine)dichlororuthenium(ii) hydrate (*cis*-[Ru(phen)_2_]Cl_2_) (98%), and potassium hexafluorophosphate (KPF_6_) (99%), tetra-*n*-butylammonium perchlorate (99%, TBAP) were purchased from Sigma-Aldrich. Dichlorvos (Nuvan insecticides) was purchased from the local market in Shillong, Meghalaya, India. The chloroform (99%), chitosan (CS) (extra pure with a viscosity of 100 mPas), silver nitrate (AgNO_3_, 99.5%), polyvinylpyrrolidone (PVP, MW = 40 kDa), ethylene glycol (99%), sodium chloride (NaCl) (99.9%), potassium chloride (99.5%), acetic acid (99.5%) were purchased from SRL, India. Milli-Q deionized water with pH = 6.5, and resistivity = 18.5 MΩ cm was used for the LB experiment. Phosphate buffer solution (pH 7) was made and used in all the sensing studies. The chemical structures of the constituent chemicals of the nanocomposite and pesticide analyte have been presented in Fig. 1A.

### 2.2 Synthesis of ruthenium polypyridine (RuPo) complex

The analyte-targeting ligand, 4,7-dihydroxy-1,10-phenanthroline (L), was synthesized following the reported procedure.^51^ The starting ruthenium complex, *cis*-[Ru(phen)_2_Cl_2_] was synthesized using the reported literature procedure^52^ and used for the preparation of RuPo complex. *Cis*-[Ru(phen)_2_]Cl_2_ (0.36 mmol, 0.192 g) and L (0.44 mmol, 0.094 g) were taken in 2 necks round-bottom flask, then 10 mL of degassed ethylene glycol was added and refluxed at 140 °C for 12 hours under nitrogen (N_2_). The reaction progress was monitored by thin-layer chromatography (TLC) and after the completion of the reaction, it was cooled down to room temperature. In silica TLC, a new spot was generated at lower *R*_f_ value than starting materials, corresponding to *cis*-[Ru(phen)_2_Cl_2_], 4,7-dihydroxyphenathroline when eluted with aqueous acetonitrile (CH_3_CN : H_2_O : saturated KNO_3_; 90 : 8 : 2) and visualized under UV light. The column chromatographic separation was done and the RuPo complex was characterized by NMR and mass spectroscopy (Fig. S1–S3, ESI†). The reaction mixture was treated with an aqueous potassium hexafluorophosphate (KPF_6_) (0.400 g) solution and stirred at room temperature for 1 hour. The dark red precipitate was collected through filtration. The crude product was purified on silica gel (CH_3_CN : H_2_O : KNO_3_ = 90 : 8 : 2). The organic solvent was evaporated and the water fraction was treated with excess KPF_6_. The pure red-colored solid ruthenium(ii) complex (RuPo, molecular weight 693 g mol^−1^) was obtained in a good yield (0.214 grams, 60%). We have characterized the RuPo complex with ^1^H, ^13^C NMR (Bruker Avance II (400 MHz) and Mass spectrometry (Waters ZQ-4000) which are sufficient evidence for the characterization of the RuPo complex (Fig. S1–S3†).

### 2.3 Preparation of chitosan (CS), silver nanowires (AgNWs), and octadecylamine (ODA) solutions

The CS solution with a concentration of 0.1 M was prepared using acetic acid (0.1 M) solvent with continuous stirring for 48 hours. The silver nanowires (AgNWs) were prepared using a polyvol process as per earlier report.^53,54^ ODA solution (1 mM) was prepared by mixing ODA in chloroform solvent with stirring.

### 2.4 Cleaning of LB trough, substrate, and electrode

The LB trough was filled with Milli-Q water and left for 2 hours for the leaching of dust and then cleaned with acetone, chloroform, and kimtech tissue paper. The water subphase was cleaned by an aspirator vacuum pump, up to a surface pressure of <0.1 mN m^−1^. All substrates (glass, quartz, and silicon wafer) and electrodes (working, counter, and reference) were cleaned with soap solution, acetone, and water using ultrasonication. The substrate and electrodes were kept in a desiccator for drying and further experiments. The hydrophilicity of the slide was confirmed by a homogeneous coating of water on the slide.

### 2.5 Langmuir Blodgett (LB) composite film preparation and electrode modification

A computerized LB film deposition instrument (model APEX LB-2007DC, Apex Instruments Co. India) was used for monolayer study and LB film preparation. The surface pressure on the water subphase of the LB trough was measured using a Wilhelmy-type balance with an accuracy of 0.01 mN m^−1^. The trough dimensions were width (130 mm), length (330 mm), and depth (20 mm). The subphase was prepared with deionized Milli-Q water with pH of 6.8 and resistivity of 18.2 MΩ cm. All experiments were carried out at a temperature of 20 ± 5 °C and reproducibility has been ensured by at least three independent runs.

The nanocomposite LB film, composed of CS + AgNWs + RuPo + ODA, was prepared by spreading individual solutions (CS, AgNWs, RuPo, ODA) on a water subphase within the surface pressure limit of 0.5 mN. A reaction time of 30 minutes was given for stabilizing the composite monolayer, and the monolayer was transferred to the substrate with lifting speed and dipping speed of 3 mm min^−1^ and 4 mm min^−1^, respectively. The pressure-area isotherm of the pure and composite monolayer was recorded. The role of different components have been investigated with the help of isotherm studies.^55–57^

The substrate and electrode were dipped into the water subphase before monolayer preparation. The LB parameters regarding film length, slide perimeter, dipping speed, lifting speed, and drying time for LB experiments were selected as per our earlier report.^58,59^

### 2.6 Preparation of composite Langmuir monolayer and Langmuir–Blodgett film

To prepare the composite monolayer on the water subphase of a trough, initially, all the components of the nanocomposite solution were prepared as described in the earlier Section 2.3. The experiment was carried out at room temperature (20 ± 5 °C). The composite monolayer was compressed with the parameters given in section 2.4. To prepare the Langmuir–Blodgett film on different substrates (Pt, quartz, silicon wafer), the substrate was immersed in the water subphase and after that, the nanocomposite solution was spread on the water trough. The drying time for above and below the water subphase was kept as 5 minutes, and 1 minute respectively. The drying time after the 1st and 2nd layer was kept as 20 minutes so that the deposition of the first layer is strong on the substrate.

### 2.7 Composite film characterization

The absorption spectra were recorded using a UV-visible spectrometer (Hitachi, Model: U-3900) with wavelength range 200–800 nm, scan speed of 300 nm min^−1^, and optical path length 10 mm. The UV-visible spectra of nanocomposite LB film were recorded on a rectangular-shaped quartz substrate. The FTIR spectra of powder and composite film were recorded using an FTIR spectrometer (Bruker, Model: ALPHA with OPUS 7.5 software) on a silicon wafer substrate.

The imaging of the composite films deposited on a glass slide was carried out using SEM (Zeiss, Model: AG-ULTRA 55) with an operating voltage of 20 kV. The film samples were mounted on brass stubs (30 mm diameter, 10 mm height) using double-sided adhesive tape. The gold coating was made on the film by a gold sputter (JFC 1100, JEOL) to increase the conductivity and to protect the film from burning effect of the electron beams.

### 2.8 Electrochemical measurements for sensor

The different electrochemical parameters have been optimized for efficient pesticide sensing, as per literature and experimentation such as scan rate at 0.1 V s^−1^.^60–62^ The electrolyte concentration was selected as per literature, such as KCl (0.1 M),^63^ PBS + KCl (0.1 M),^64^ and TBAP (0.1 M).^41^ All parameters for Differential Pulse Voltammetry (DPV) are aligned with those used in the literature for pesticide sensing: initial *E* (V) = 0.8, final *E* (V) = 1.6, increment *E* (V) = 0.004, amplitude (V) = 0.05, pulse width (s) = 0.06, sample width (s) = 0.02, pulse period (s) = 0.5, and quiet time (s) = 2.^65^ Additionally, the Square Wave Voltammetry (SWV) parameters were set as follows: initial *E* (V) = 0.8, final *E* (V) = 1.6, increment *E* (V) = 0.004, amplitude (V) = 0.025, frequency (Hz) = 10, and quiet time (s) = 2.^66^ These parameters are consistent with those used in the literature for pesticide sensing.

## 3. Result and discussion

### 3.1 Study of composite isotherm and characterizations

#### 3.1.1 Study of isotherm and surfactant optimization

**Fig. 2:**
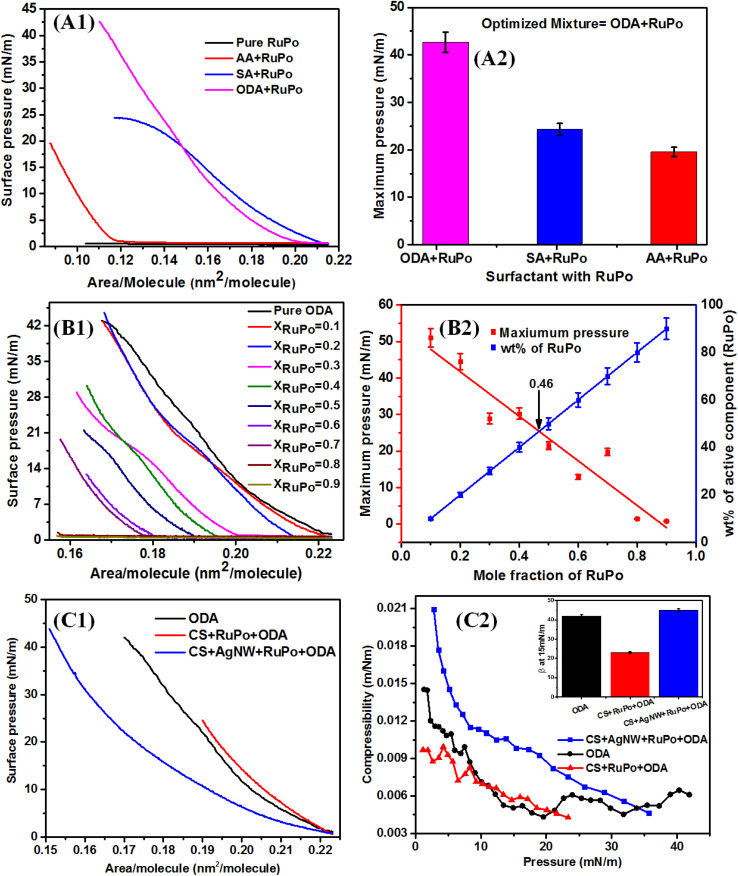
(A1) Isotherm of different surfactants mixed with RuPo (1 : 1 ratio), (A2) bar diagram for maximum pressure of different monolayers, (B1) Isotherm of ODA monolayer with different mole fractions of RuPo, (B2) plot of maximum pressure, and wt% of the active compound with the variation of mole fraction of RuPo. (C1) Isotherm of ODA, CS + RuPo + ODA, and CS + AgNWs + RuPo + ODA monolayers. (C2) Compressibility plot obtained from π–A isotherms. Inset represents the compressibility bar plot at lifting surface pressure (15 mN m^−1^).

The π–A isotherm of nanocomposite monolayer formed at the air/water interface contains gaseous (G), liquid (L), and condensed (C) phases of monolayer.^67^ Several features are seen from the isotherm as shown in Fig. 2(A1 and C1). The “lift-off area” in Langmuir–Blodgett (LB) experiments refers to the surface pressure or area per molecule at which a monolayer begins to form on the surface of a liquid, marking the transition from a dispersed to a condensed phase.^68^ In case of CS + AgNWs + RuPo + ODA monolayer, the changes in lift off area from 0.225 nm^2^ to 0.212 nm^2^, due to G-L transition. The gas–liquid (G-L) transition in a Langmuir isotherm describes the phase change from a gas-like, dispersed state to a liquid-like, condensed state in a monolayer, marked by a distinct change in the slope of the surface pressure *vs.* area per molecule.^69^ The increasing part of compression up to an upper limit of π = 20 mN m^−1^, is due to the L state of monolayer. Similar nature of the G–L transition and the existence of the L state was found in the case of RuPo + ODA. Further compression of the system between π = 20–30 mN m^−1^, a plateau is observed due to the partial squeeze-out of the nanocomponents from the monolayer preceding the full collapse.

The influence of different phases on the properties and applications of a monolayer is significant. In general, monolayer properties are different in different phases such as gaseous (G), liquid (L), and condensed phase. In G phase, the molecules are randomly distributed with minimal interaction, resulting in low surface pressure and a lack of organization, which cannot form a film.^70^ In the L phase, molecules are closely packed yet still keeping a liquid-like state, making it suitable for applications like certain types of coatings but not recommended for film.^70^ The condensed phase (C) exhibits tightly packed and well-organized molecules with high surface pressure, ideal for creating stable and reproducible films necessary for sensors.^71^ The observed plateau between π = 20–30 mN m^−1^ indicates that the monolayer has achieved maximum adsorption. The monolayer becomes more stable and the tight packing of molecules enhances the monolayer's mechanical strength as well as other material properties for various applications such as sensors.^72^ The composite films have been lifted before the plateau region at 15 mN m^−1^.

To prepare the LB film of the RuPo complex, the solution of RuPo with acetonitrile solvent was spread on the air/water interface. However, the surface remains close to zero due to the non-amphiphilic character of RuPo (Fig. 2A1). The ODA also has been optimized among three anionic, cationic and neutral surfactants (ODA, SA, and AA) through Langmuir monolayer isotherm study (Fig. 2A1 and A2). The surface pressure-molecular area (π–A) isotherm of the RuPo and ODA mixture exhibited the highest surface pressure compared to those of AA or SA. Therefore, the ODA + RuPo mixture was selected for further studies, as illustrated in the bar diagram (Fig. 2A2). The isotherm curves are in Fig. 2A1, shows the variation of x-value of the pressure-area isotherm of different surfactants ODA, AA, and SA. The lift-off x-value of an isotherm represents the molecular area per molecule, which closely matches with the hydrodynamic dimension of the surfactant molecule under the interactions at the air–water interface.^73^ This difference in molecular weight of different surfactant such as ODA (259 g mol^−1^), AA (312 g mol^−1^), SA (284 g mol^−1^), leads to difference in their dimension as well as their chemical structure may affect the packing and orientation, which resulted in the isotherm difference. Fig. 2(B1 and B2) shows the wt% optimization of RuPo in ODA + RuPo mixed monolayer, as discussed later.

Fig. 2C1 shows the surface pressure-molecular area (π–A) isotherm of pure ODA, CS + RuPo + ODA composite, and CS + AgNWs + RuPo + ODA composite monolayers. The lifting area/molecule (nm^2^ per molecule) of pure ODA and pure CS monolayer (Fig. S4†) was found to be 0.12 nm^2^ and 0.2 nm^2^, respectively, which are in line with the earlier reports.^74,75^ The shifting of the composite isotherm compared to pure ODA and CS, indicates the formation of composites at different extent. Fig. 2C2 indicates the maximum surface pressure achieved by CS + AgNWs + RuPo + ODA composite monolayer along with ODA and CS + RuPo + ODA, respectively. However, CS + AgNWs + RuPo + ODA composite has been optimized due to its better surface pressure performance in the monolayer as well as better sensor current delivered. Here AgNWs act as an electron transfer enhancer and the CS + AgNWs + RuPo + ODA nanocomposite film has been used to modify electrodes for further detection of pesticide (BF) using the electrochemical technique, as discussed in a later section.

#### 3.1.2 Optimization of the molar fraction of electrochemically active compound (RuPo complex) in Langmuir monolayer

Fig. 2B1 shows the isotherm of ODA + RuPo mixed monolayer with different molar ratios of RuPo. Fig. 1B shows the schematic representation of the formation of a composite monolayer on the air/water interface using the LB technique. Fig. 2B2 shows the bar diagram of the maximum surface pressure achieved by different ODA + RuPo mixed monolayers. The wt% of RuPo has been experimentally optimized by varying the wt% of RuPo in the isotherm study (Fig. 2B2). The intersection point between the percentage of the active component and the maximum pressure achieved using the different mole fraction of RuPo is found to be at *X*_RuPo_ = 0.46 as seen in Fig. 2B2. The ODA + RuPo mixed monolayer with 46% RuPo (*X*_RuPo_ = 0.46) has been qualitatively selected as an optimized mixed monolayer for sensor application (Fig. 2B2). An increased amount of RuPo negatively impacts the ODA monolayer, preventing it from reaching sufficient surface pressure to enable multiple transfers onto a substrate and less amount of RuPo in the LB film will impact in the voltammetry signal, since RuPo is the electrochemically active compound, as discussed in the later section also. Hence wt% optimization of RuPo will help in achieving higher sensing performance. The wt% of the different components in the composite were CS 70%, RuPo 20%, AgNWs 5% and ODA 5%. The synergistic role of RuPo and AgNWs on the film preparation and BF pesticide detection have been reflected in different data as discussed later sections. The ODA surfactant played the role in LB film preparation as a floating lipid matrix along with chitosan as a biocompatible polymer matrix, ruthenium complex as an electrochemically redox-active compound, and AgNWs nanocomponent as electron transfer enhancer^55–57^ (Fig. 1B).

#### 3.1.3 Compressibility study of composite Langmuir film

To specify the phase change during compression, we performed a surface compressibility analysis on the Langmuir monolayer. The compressibility investigation is critical for understanding the phase change behavior as well as the mechanical stability of the monolayer at the air/water interface.^76^ The β–π was studied for the different composites monolayer as shown in Fig. 2C1. The β–π curve in a Langmuir isotherm illustrates the relationship between compressibility (*β*) and surface pressure (π) of a monolayer on a liquid surface, showing highest compressibility at low surface pressures (gas-like phase) and decreasing compressibility as the monolayer transitions to liquid-expanded and then to liquid-condensed or solid-like phases.^55^ The compressibility coefficient (*β*) is computed using the eqn (1).^76^1
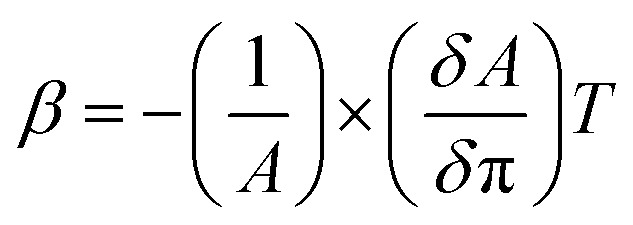
where, *β* is the compressibility coefficient, *A* is the area/molecule and the appearance of any peaks in the *β*–π curve can represent some transitions, and the peak heights indicate the maximum compressibility (*β*_max_) of the monolayer.^77^

The *β*–π curve (Fig. 2C2) obtained for the composite (CS + AgNWs + RuPo + ODA) shows no prominent peak indicating no phase transition in the monolayer. The maximum compressibility for the ODA and CS + RuPo + ODA was found to be at *β*_max_ = 0.014 m mN^−1^ and *β*_max_ = 0.009 m mN^−1^ respectively, at film lifting pressure (15 mN m^−1^). The mechanical strength, or maximum compressibility, of the composite film (CS + AgNWs + RuPo + ODA) increased by 2.2 times when AgNWs were added. The compressibility value rose from *β*_max_ = 0.009 m mN^−1^ (without AgNWs) to *β*_max_ = 0.020 m mN^−1^ (with AgNWs), as shown in Fig. 2C2. Thus, both the CS and AgNWs components contributed to the enhanced mechanical stability of the composite monolayer.

#### 3.1.4 UV-vis spectroscopy characterization for different layer LB film

**Fig. 3:**
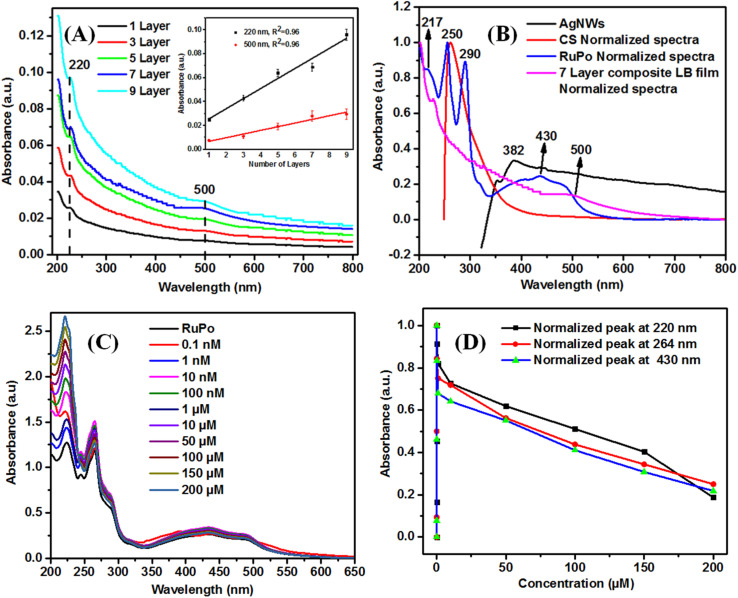
(A) UV-visible spectra of composite LB film (CS + AgNWs + RuPo + ODA) with different layer number (1, 3, 5, 7, and 9 layers), inset shows linear fitting of absorption intensity taken at 220 nm and 500 nm, with different layer number (1, 3, 5, 7, 9), (B) UV-visible spectra different component of LB film (AgNWs, CS, and RuPo), (C) UV-visible spectra of RuPo mixed with different concentration of pesticide (BF), (D) graph showing absorbance *versus* concentration of pesticide (BF) at different peaks.

Fig. 3A shows the UV-visible spectra of CS + AgNWs + RuPo + ODA composite multilayer LB film with different layers (1, 3, 5, 7, and 9). Fig. 3A inset shows the linear fitting of absorption intensity taken at 220 nm and 500 nm, with layer numbers. Fig. 3B shows the UV-visible spectra of different components of LB film such as AgNWs, CS, and RuPo. The absorbance peaks at 383 nm and 250 nm, correspond to the silver nanowires and chitosan, respectively.^78,79^ The absorbance peaks at 290 nm is assigned to the intraligand (IL) π–π* transitions of phen/bpy (ligand) of RuPo.^41^ The absorbance peak at 430 nm corresponds to the metal-to-ligand charge-transfer (MLCT) transition of RuPo.^43^ The absorption spectra of LB composite film contain a peak at 220 nm and 500 nm, which may originate from the π–π* transition of RuPo and MLCT transition of RuPo, respectively.^41^Fig. 3B shows an absorption peak at 500 nm for the composite LB film, which may be due to the aggregation of silver triangle, cube, and nanowire in the LB film, and also accompanied by the charge transfer band of RuPo.^80^ The correlation coefficient of linear fitting for the peak 220 nm and 500 nm was achieved as *R*^2^ = 0.96 and *R*^2^ = 0.96 respectively, which indicates that the layer-by-layer film was transferred linearly on the quartz substrate without any loss of material.

#### 3.1.5 Interaction of active compound (RuPo) and pesticide (BF) target analyte

The interaction mechanism of BF pesticide with RuPo compounds, have been studied using UV-visible spectroscopy (Fig. 3C). We have taken the concentration of pesticide from nM to μM and mixed it with the active RuPo compound. Fig. 3C shows the changes in the corresponding peak with the different concentrations. The peaks at 220 nm and 264 nm correspond to the π–π* transitions of phen/bipy (C_5_H_5_N) and 4,7-dihydroxy-1,10-phenanthroline (L) (–OH) ligands and the peak at 430 nm corresponds to the metal-to-ligand charge-transfer transition band. Fig. 3D shows the trend of the three peaks with concentration, which initially increased from 0.1 nM to 10 nM and subsequently decreased from 10 nM to 200 μM. The increase in absorbance could indicate that the pesticide interacts with the active compound, where the reducing group of –OH or –NH in BF pesticides readily reacts with the oxygen-containing RuPo compound.^81^ Conversely, a decrease in absorbance might suggest that the pesticide is interfering with the active compound in a way that reduces its ability to absorb light at the measured wavelength due to the breakdown or degradation of the active compound.^54^ The increase and decrease nature of UV-vis spectra is seen in Ru metal containing complex, which seems to be common observation in other metal complex also like Fe containing porphyrin in hemoglobin.^54,57^ Also, the phenolic –OH groups of the 4,7-dihydroxy-1,10-phenanthroline ligands of the RuPo can form hydrogen bonds with the carbonyl (C=O) or ester groups (R–CO–OR) in bifenthrin,^82^ which is also reflected in the shifting of the CV and DPV peak position in the presence of pesticide.^83^ Additionally, both 4,7-dihydroxy-1,10-phenanthroline and bifenthrin contain aromatic rings, which engage in π–π stacking interactions, further stabilizing the complex.^84^ Schematic mechanism for interaction of the RuPo with the BF pesticide is shown in the Fig. S5.†

#### 3.1.6 FTIR, SEM, and TEM characterization

**Fig. 4:**
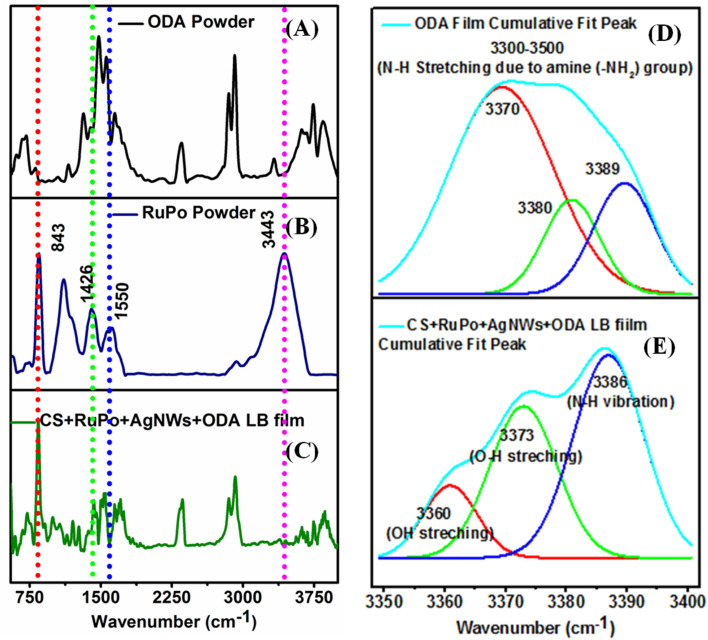
(A–C) FTIR spectra of ODA powder, RuPo powder, and CS + AgNWs + RuPo + ODA LB film, respectively. (D and E) Deconvoluted FTIR spectra of pure ODA LB film and CS + AgNWs + RuPo + ODA LB film, respectively.

Fig. 4A–C shows the FTIR spectra of ODA in powder, RuPo in powder, and RuPo nanocomposite film, respectively. The FTIR spectra of the composite indicate the ODA, AgNWs, and CS are in line with earlier reports.^54,85^ There is a change in the relative intensities in the region 1400–1680 cm^−1^ (C=C stretching) from RuPo powder to RuPo film due to the lesser amount of compound in the film.^86,87^ The peaks at 843 cm^−1^ (P–F stretchings), 1426 cm^−1^ (C=N stretching), and 3443 cm^−1^ (N–H stretchings) are the characteristic peaks of the RuPo which are also present in the composite LB film.^41^ The IR peaks in the composite at 2353 cm^−1^ (C=O stretching vibrations), 3742 cm^−1^ (hydroxyl –OH group of polymers), and 3843 cm^−1^ (N–H stretching vibration) are due to the presence of ODA, and chitosan, respectively.^54^ Table S1† shows the summary of the FTIR peak assignment for composite LB film. Fig. 4(D and E) show the deconvoluted spectra of the ODA film and the RuPo composite LB film, respectively, in the range of 3500–3400 cm^−1^. The peaks at 3370 cm^−1^, 3380 cm^−1^, and 3389 cm^−1^ in the ODA film rise due to the N–H stretching of the amine (–NH_2_) group of ODA as well as ODA on the RuPo surface (Fig. 4D).^87,88^Fig. 4E, the peak at 3360 cm^−1^ (OH stretching), is the characteristic peak of chitosan.^89,90^ The broadening of the peak at 3373 cm^−1^ (O–H stretching) is due to the attachment of ODA on the RuPo surface (Fig. 4E).^87,91^ Also, the peak at 3386 cm^−1^ from N–H stretching vibration is due to the presence of RuPo and 3743 cm^−1^ corresponds to –OH group of chitosan (Fig. 4E).^92,93^

**Fig. 5:**
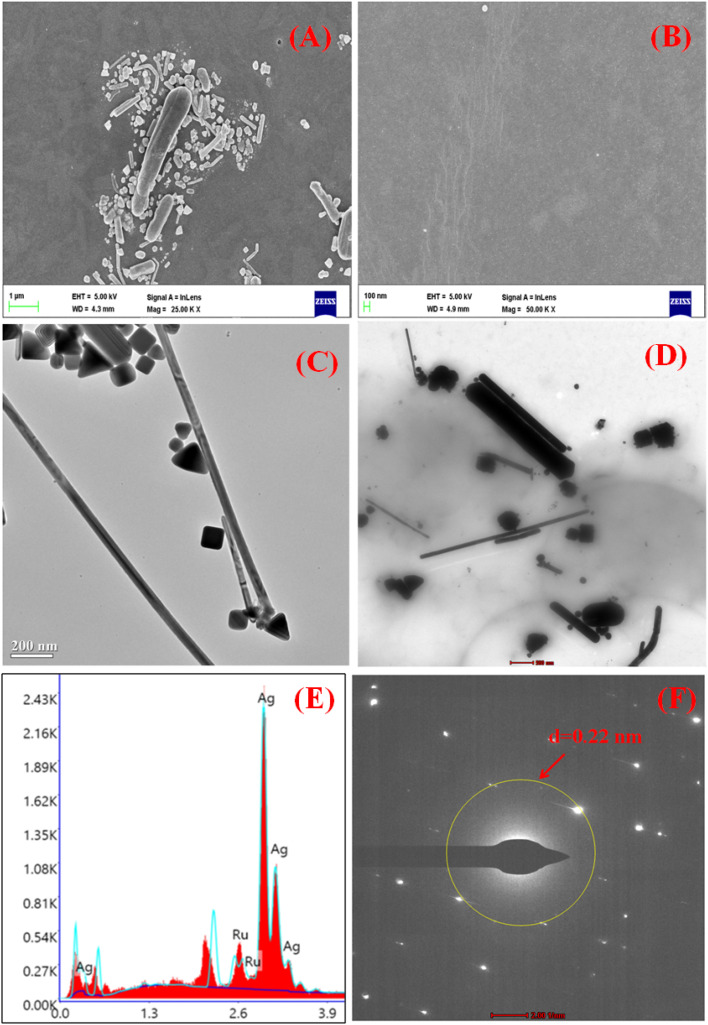
(A and B) SEM image of LB composite film in μm and nm scale, (C and D) TEM image of AgNWs with and without pesticide interaction, (E and F) EDX and SAED pattern analysis, respectively.

Fig. 5(A and B) show the SEM images of the nanocomposite LB film at 1 μm scale and 100 nm scales, respectively, which confirms the formation of a uniform composite monolayer containing a ruthenium complex on the glass substrate. The average diameter and length of the AgNWs were found to be 50 nm and 5 μm respectively, which is also reported in previous work.^54^ The TEM characterization has also been performed to study the pesticide interaction with silver nanowires. Fig. 5(C and D) shows the TEM image of AgNWs without pesticide interaction and with pesticide interaction, respectively. The confirmation of ruthenium (Ru) and AgNWs in the nanocomposite film was achieved using energy dispersive X-ray spectroscopy (EDX), as demonstrated in Fig. 5E. The d-value from the SAED pattern (Fig. 5F) is calculated as 0.225 nm which is in line with literature report.^94^

When BF (10 μM) was mixed with the AgNWs solution, the aggregation of the nanowires took place as shown in Fig. 5D. The interaction may be due to the presence of chemical groups in pesticides that interact with the surface of the nanowires.^95^ This interaction could lead to the formation of chemical bonds or attractive forces that cause the nanowires to aggregate.

### 3.2 Electrochemical sensing of pesticide

#### 3.2.1 Electroactivity optimization of RuPo by electrolyte, pH, and AgNWs

**Fig. 6:**
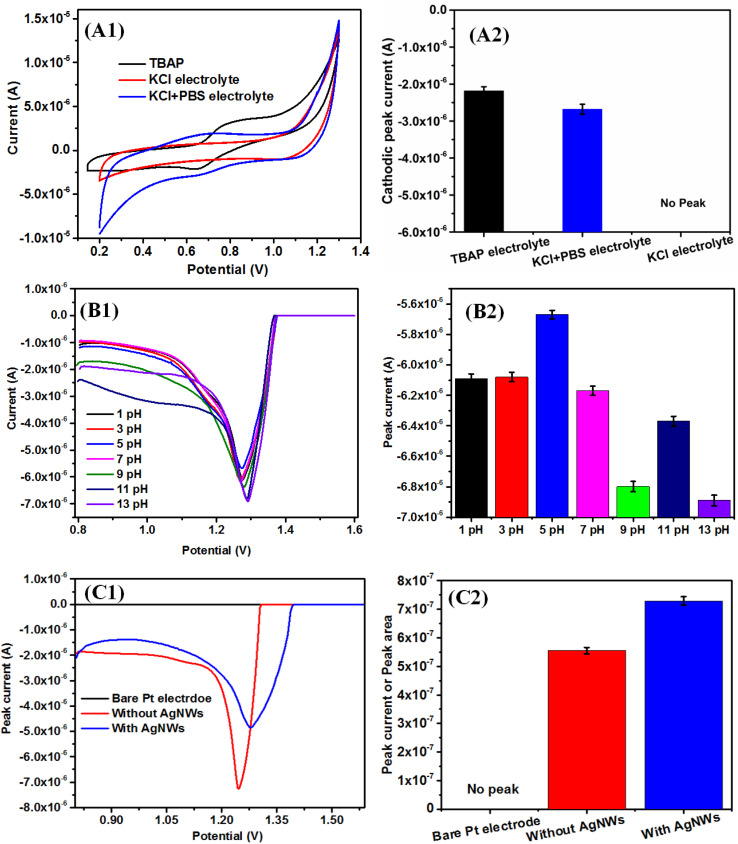
(A1) CV graph of Pt modified electrode using different electrolytes (A2) bar diagram for the different electrolyte *versus* current, (B1) DPV variation of the different pH (1–11), (B2) bar diagram for the different pH *versus* current (10 μM). (C1) DPV variation of Pt modified electrode in the presence of AgNWs, (C2) Bar diagram for the presence of AgNWs on Pt modified electrode *versus* current.

The CS + AgNWs + RuPo + ODA composite LB film-modified Pt electrode have been tested for cyclic voltammetric current signals using different electrolytes such as tetra-*n*-butylammonium perchlorate (TBAP), KCl and KCl + PBS (Fig. 6A1) with a potential window of 0.2–1.3 V. The cathodic and anodic peak of RuPo is found at 0.99 V and 1.05 V using TBAP as electrolyte, respectively.^41^ The cathodic and anodic peak of RuPo shifted to 0.65 V and 0.82 V in CS + AgNWs + RuPo + ODA composite modified electrode (Fig. 6A1), due to the changes from RuPo in solution to RuPo in the LB film.^96^Fig. 6A2 shows the cathodic peak current response of the composite modified electrode in TBAP, KCl + PBS as 3.28 × 10^−6^ A and 1.85 × 10^−6^ A, respectively. However, there is no observable peak in case of KCl electrolyte. The TBAP electrolyte produces a better current signal than KCl + PBS or KCl, still KCl + PBS (0.1 M) has been selected as an optimized electrolyte due to its low cost in order to develop a cost-effective electrochemical pesticide sensor. The CV technique has been used to select electrolytes. However, the selected electrolyte (KCl + PBS) does not produce a prominent redox peak of RuPo in the film by CV technique. Hence, pulse voltammetric techniques like SWV and DPV, which produce a clear peak at 1.29 V of RuPo, have been adopted for further sensing studies. The shift in cathodic peak potential is due to the difference of both techniques in CV *vs.* DPV. The CV cathodic peak appeared as a broad peak, which comprises both faradaic and non-faradaic currents.^97^ On the other hand, DPV is a pulsed technique and highly sensitive, which minimizes the non-faradaic component of current.^97^ The height of the peak current in DPV is directly proportional to the concentration of redox active species in solution. The shift in cathodic potential value from 0.82 V to 1.29 V, may be due to the above-mentioned contribution of currents as well as may be due to the presence of active compound RuPo in the nanocomposite.

The DPV and SWV are very sensitive and allow direct analysis of analytes at the ppb (parts per billion) and even ppt (parts per trillion) level.^54,98^Fig. 6B1 shows the DPV plot with the variation of pH (1–11) using KCl + PBS electrolyte. The pH was adjusted by HCl and NaOH, and PBS was used to maintain the pH condition. The concentration of the BF pesticide used was 10 μM. The observed maximum current at pH = 5 signifies the protonation reaction in the electrochemical set up, which may be supported by the slope value of pH *vs.* peak potential graph. In our earlier study also indicates that the phenolic –OH of the pure RuPo complex shows higher electrochemical activity as the pH increases, due to the formation of the phenoxide ion.^41^ The present study with RuPo composite, the observed slope value in ithe pH range 1–7 is −1 mV pH^−1^, indicating the reaction protonation mediated and it is +3.3 mV pH^−1^ in the pH range 7–13, indicates deprotonation mediated as per Nernst theory^99^ (Fig. S6†). The observed maximum peak current at pH = 5 indicates the electrochemical process to be protonation mediated, which is in line with earlier reports on pK value at 5.^99,100^ This shows that weak acidic environment is appropriate for electrode process involved in proton transfer.^101^

Fig. 6C1shows the DPV curve of bare electrode, modified electrode without AgNWs, and with AgNWs, respectively. Fig. 6C2 shows the bar plot of DPV peak area *versus* the different modified electrode. The peak current is more accurately measured by peak area compared to peak height, which is proportional to the efficient redox process at the electrode.^102^ In the composite electrode, the area of the curve with AgNWs is 7.29 × 10^−7^ and without AgNWs is 5.55 × 10^−7^ (Fig. S7†). We observe that the area of the curve with AgNWs is 1.31 times higher compared with the area of the curve without AgNWs which means the higher electroactivity of the composite with AgNWs.

#### 3.2.2 Study of selectivity and other sensing parameters

**Fig. 7:**
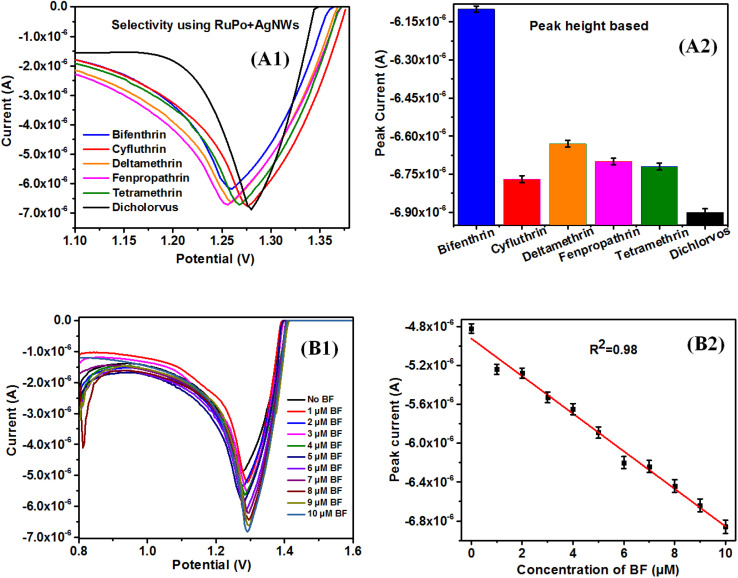
(A1) DPV graph of the composite (CS + AgNWs + RuPo + ODA) modified electrode with different pesticides. (A2) Selectivity study with bar plot of peak current *versus* different pesticides. (B1) DPV graph of composite modified Pt electrode with different concentration of bifenthrin, (B2) calibration curve with analyte (bifenthrin pesticide) concentrations *vs.* peak current.

The selectivity study with other relevant pesticides, have been conducted such as some pyrethroid pesticides (bifenthrin, cyfluthrin, deltamethrin, fenpropathrin, tetramethrin) as well as dichlorvos (organophosphate group) (Fig. 7A1). The dichlorvos have been used in the selectivity study, since the same RuPo compound has been reported earlier to detect dichlorvos by fluorescence technique^41^ and the present sensor by electrochemical method and using RuPo based composite. As per literature report, electrochemical signals can be used for selectivity studies in different methods such as peak area, peak height, peak FWHM^102–104^ and peak magnitude.^105^ The selectivity study basically needs to use some method to select the targeted pesticide, which discriminates from the other relevant pesticide and interfering agents. In the present work, the selectivity has been analysed by peak height (Fig. 7A2), peak area (Fig. S8A†), FWHM (Fig. S8B†), peak magnitude (Fig. S8C†). It is found that only the peak height method can better discriminate BF from others and can show selectivity compared to other methods (peak area, peak magnitude, FWHM). The area calculation from the DPV data, are presented in Fig. S9(A–F).†

Therefore, bifenthrin shows better selectivity and was selected as a target sensor analyte for the determination of other parameters like sensitivity, detection limit, *etc.* It is to be noted that the selectivity of RuPo in the solution phase has been reported for dichlorvos pesticide,^41^ however, present reports of RuPo-based composite film have selectivity with BF pesticide. The difference in the selectivity of RuPo may be due to its presence in the complex nanocomposite LB film, where the nanocomponent AgNWs may contribute in tuning the selectivity of the parent RuPo compound. We have selected the CS + AgNWs + RuPo + ODA Pt modified electrode because it showed higher selectivity towards BF pesticide over other pesticides.

Fig. 7(B1 and B2) shows the DPV data of the modified electrode in the range 0.8 to 1.6V with varying concentrations of BF pesticide. Fig. 7B2 shows the calibration curve of BF pesticide sensors using DPV techniques. Fig. S10A1 and B1† shows the SWV and CV data of the modified electrode with varying concentrations of BF pesticide, respectively. Fig. S10A2 and B2† show the calibration curve of BF pesticide sensors using DPV and CV techniques, respectively. The sensitivity has been calculated using the slope of the calibration graph and the working area of the electrode using eqn (2).^106^ The area of the Pt working electrode was calculated using slide calipers and was found to be 0.2826 cm^2^, excluding the PTFE coating. The sensitivity of BF pesticide is found to be 0.546 μA cm^−2^ μM^−1^, 0.648 μA cm^−2^ μM^−1^, and 0.291 μA cm^−2^ μM^−1^ using SWV, DPV, and CV technique, respectively. This is a single reported data on BF pesticide using an electrochemical technique. The sensing parameters of the present BF pesticide sensor and BF detection parameters by other methods are shown in Table 1.2
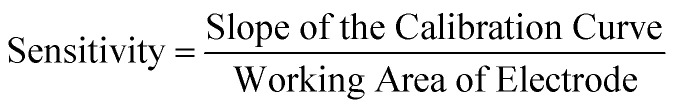


**Table 1:** Sensing data of the reported BF sensor and literature data using different techniques^a^

Sl. No	Method	Sensitivity	LOD	LR	Reference, year
1	Electrochemical(DPV)	0.648 μA cm^−2^ μM^−1^	1 μM	1–10 μM	Current work
	Electrochemical (SWV)	0.546 μA cm^−2^ μM^−1^	1 μM	1–10 μM	
	Electrochemical (CV)	0.291 μA cm^−2^ μM^−1^	4 μM	1–9 μM	
2	Electrochemical	NR	1.6 μg L^− 1^	4 to 50 μg L^−1^	[^107^, 2022]
3	Fluorescence	NR	0.4 μM	10–300 μM	[^22^, 2015]
4	Fluorescence	NR	0.08 μM	0.5–40 μM	[^23^, 2020]
5	Fluorescence	NR	0.02 mM	NR	[^25^, 2023]
6	SERS	NR	0.01 mM	NR	[^24^, 2023]
7	Chemiluminescence	NR	0.07 μg mL^−1^	0.11–55.5 μg·mL^−1^	[^108^, 2011]
8	ELISA	NR	9 μM	NR	[^20^, 2015]
9	ELISA	NR	5 mM	NR	[^19^, 2008]
10	ELISA	NR	50 ng mL^−1^	NR	[^109^, 2022]
11	UPCC	NR	47 μM	NR	[^18^, 2015]
12	RTP	NR	39 mM	NR	[^21^, 2017]

a ELISA = enzyme-linked immunosorbent assay, UPCC = Ultra performance convergence chromatography, RTP = room-temperature phosphorescence, SERS = surface enhanced Raman spectra.

The limit of detection (LOD) has been calculated using the slope and standard deviation (SD) of the calibration curve using eqn (3).^110^ SD is calculated in the following steps. First we have taken the mean – value and deviation was calculated from the mean followed by squaring. In the second step, we find the variance and square root of variance give the SD value. The LOD of the BF sensor is found to be 1 μM in both SWV and DPV techniques, and 4 μM using CV technique.3
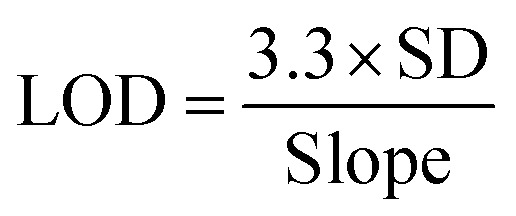


The LR value of a sensor is generally defined and calculated from the linear fitting of the calibration curve with *R*^2^ value >0.95 or within 5% deviation. The LR value of BF pesticide sensing is found as 1–10 μM (DPV), 1–10 μM (SWV), and 1–9 μM (CV) with the *R*^2^ value of the linear fitting as 0.98, and 0.98, 0.98 respectively.

#### 3.2.3 Study of stability, repeatability, interference, reproducibility, and real samples analysis

The stability of the BF sensor has been calculated using eqn (4).^111^ The peak current gradually changes in every cycle and attains saturation. The increase in peak current indicates the higher population of active sites on the Pt electrode and the decrease in peak current indicates the passivation of the electrode surface. The stability value of the modified electrode is found to be 91%, and 84% using SWV and DPV, respectively. The stability value indicates that the signal response of the modified electrode is stable enough for its repeated uses.4
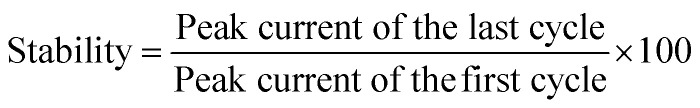


**Fig. 8:**
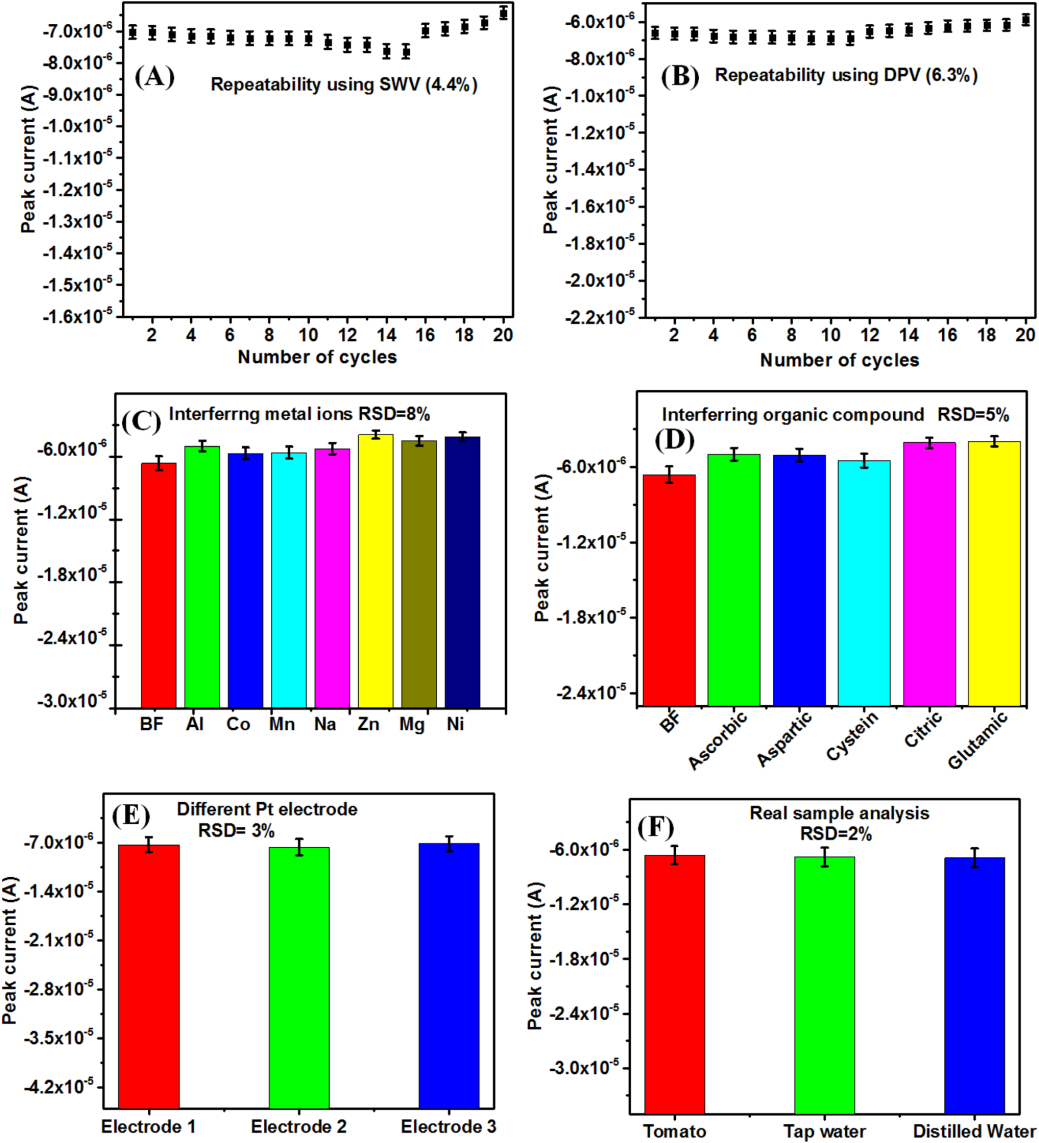
(A) Repeatability of Pt-modified electrode using DPV, (B) repeatability of Pt-modified electrode using DPV, (C) interference study of metal ions using Pt-modified electrode, (D) organic compounds, (E) reproducibility of DPV response for different LB film modified Pt electrodes in the presence of 10 μM BF pesticide, (F) comparison of the peak current of LB film modified Pt electrode in real samples (tomato, tap water, distilled water) and in the presence of 2 μM BF pesticide.

Fig. 8(A and B) shows the repeatability study up to 20 cycles of BF pesticide sensing using SWV and DPV techniques, with RSD value of 4.4% and 6.3%, respectively. This indicates the sensor developed by LB film-modified electrode, can give reliable and repeatable data. The different LB films can give repeatable data due to the fact that the LB film thickness can be computer controlled by its layer number. The interference studies of the reported BF pesticide sensor have been carried out in the presence of 2 μM concentration of different metal ions (Zn, Co, Ni, Al, Mg) as well as 2 μM different organic interfering species (ascorbic acid, aspartic acid, glutamic acid, citric acid, glycine) along with 10 μM of BF pesticide (Fig. 8(C and D)). It is found that the interfering species could change the DPV response of LB film-modified Pt electrodes only up to an RSD value of 8% and 5% in the presence of metal ions and organic interfering species, respectively. This reveals the good consistency of sensor response in the presence of interfering agents.

The reproducibility of the reported sensor has been carried out (Fig. 8E) using 3 numbers of LB film-modified electrodes. It is found that the RSD value from 3 electrode responses is 3%, which represents the reported sensor to be reproducible. As per, WHO and Codex Alimentarius International Food Standards, the RSD value should be < 20% for the determination of pesticide residues in food samples.^112^ The reported ACM pesticide sensors have been tested for real sample analysis in the case of tomato, distilled water, and tap water in the presence of 2 μM BF pesticide (Fig. 8F). The BF pesticides have been widely used in tomato farming and hence it is used in real sample analysis.^113^ The RSD for real sample analysis is found to be 2%, which is much less than the WHO recommendation. The different sensing parameters such as (sensitivity, LOD, LR, repeatability, reproducibility, stability, and interfering response) have been reported, which are lacking by other methods in the literature available for BF pesticide detection.

## 4. Conclusion

An electrochemical pesticide sensor has been developed to selectively detect a BF pesticide using SWV, and DPV techniques with sensitivity of 0.546 μA cm^−2^ μM^−1^ and 0.648 μA cm^−2^ μM^−1^ respectively, using a novel CS + AgNWs + RuPo + ODA composite LB film modified electrode. To the best of our knowledge, the sensing study of the BF pesticide has not yet been explored using nanocomposite for electrochemical technique. The selectivity of the BF sensor is due to the presence of the metal organic complex (RuPo) in the composite LB film. The formation of the composite CS + AgNWs + RuPo + ODA, was studied by the surface-pressure isotherm using the LB technique as well as UV-visible spectroscopy, SEM, and FTIR technique. The performance of RuPo, has been optimized in LB film by varying surfactant, pH, wt% of RuPo, AgNWs nanofiller, and electrolyte. The redshift at 500 nm in the UV-visible spectra of composite LB film is due to the charge transfer band of RuPo and also due to the aggregated presence of AgNWs in the composite LB film. The presence of characteristic peaks of the individual nanocomponent in FTIR spectra confirms the formation of composite LB film. The achieved sensing parameters such as limit of detection (LOD), linear range (LR), and sensitivity have been reported using SWV, DPV, and CV techniques. However, DPV technique showed the highest sensing performance such as sensitivity (0.648 μA cm^−2^ μM^−1^), LR (1–10 μM), and LOD (1 μM). The stability of the LB film Pt-modified electrode was 91% and 84% for SWV and DPV techniques, respectively. The sensor response is repeatable within RSD value of 4.4% and 6.3% using SWV and DPV techniques, respectively. The reproducibility of the sensor was found to be 3%. The developed sensor demonstrated BF selectivity with 2× more current compared to other pesticides and a maximum RSD value of 8% using metal ion interference, which is within WHO recommendation of 20%. The results were found to be promising as compared with other existing methods. The present study opens up new research options on highly sensitive sensor development for other different kinds of hazardous pyrethroid pesticides using LB film-based nanocomposite modified electrodes.

## Data availability

The data supporting this article have been included as part of the main manuscript and ESI.†

## Author contributions

Sanjeev Bhandari: composite LB film, electrode modification, measurements, draft manuscript; Bhaskar Sen: synthesis of RuPo complex, characterization, Snehadrinarayan Khatua: work plan, analysis; L. Robindro Singh: measurements and sample characterization, analysis; Vijay Singh Parihar: conceptualization, manuscript editing and reviewing Mrityunjoy Mahato: work plan, project handling, analysis, manuscript editing and reviewing.

## Conflicts of interest

The authors declare that there is no competing interest.

## Supplementary Material

RA-014-D4RA04188G-s001
