# Supplementary material for: Ruthenium complex based nanocomposite film with enhanced and selective electrochemical sensing of bifenthrin pesticide
Source: RSC Adv. 2024 Sep 18;14(40):29542–58. doi: 10.1039/d4ra04188g (PMC11409230; doi:10.1039/d4ra04188g)
Supplement: RA-014-D4RA04188G-s001 [file RA-014-D4RA04188G-s001.pdf]

Supplementary Information (SI) for RSC Advances.
This journal is © The Royal Society of Chemistry 2024

Supporting Information

to

Ruthenium Complex Based Nanocomposite Film with Enhanced and Selective Electrochemical Sensing of Bifenthrin Pesticide

Sanjeev Bhandari[a], Bhaskar Sen[b], Snehadrinarayan Khatua[b], L. Robindro Singh[c], Vijay Singh Parihar[d]*, Mrityunjoy Mahato[a]*

*To whom correspondence should be addressed:

[a]Physics Division, Department of Basic Sciences and Social Sciences, School of Technology, North-Eastern Hill University, Shillong, Meghalaya, 793022, India, Email: mrityunjoyedu@gmail.com

[b]Centre for Advanced Studies, Department of Chemistry, North-Eastern Hill University, Shillong, Meghalaya 793022, India

[c]Department of Nanotechnology, School of Technology, North-Eastern Hill University, Shillong, Meghalaya, 793022, India.

[d]Biomaterials and Tissue Engineering Group, Faculty of Medicine and Health Technology, Tampere University, 33720 Tampere, Finland, Email: vijay.parihar@tuni.fi

^{1}H NMR, ^{13}C NMR , and ESI-MS spectrum of RuPo complex

ESI-MS: m/z calculated for $[C_{36}H_{24}F_6N_6O_2PRu]^+$ 818.65; found: 818.67; $[C_{36}H_{23}N_6O_2Ru]^+$ 672.68; found: 672.68. 1H NMR (400 MHz, CD_3CN): δ (ppm) = 9.86 (s, 1H), 8.57 (dd, J = 8.2, 1.3 Hz), 8.54 (dd, J = 8.3, 1.3 Hz, 1H), 8.28 (s, 1H), 8.22 (d, J = 1.4 Hz, 2H), 8.15 (dd, J = 5.3, 1.3 Hz, 1H), 8.01 (dd, J = 5.2, 1.2 Hz, 1H), 7.64 (dd, J = 8.2, 5.3, 1H), 7.58 (m, 2H), 6.99 (d, J = 6.1, 1H). 13C NMR (100 MHz, CD_3CN): δ (ppm) = 162.8 (1C), 154.0 (1C), 153.8 (1C), 153.8 (1C), 149.7 (1C), 149.3 (1C), 149.1 (1C), 137.2 (2C), 131.9 (1C), 131.8 (1C), 129.0 (1C), 128.9 (1C), 126.7 (1C), 126.6 (1C), 123.9 (1C), 121.6 (1C), 111.1 (1C).

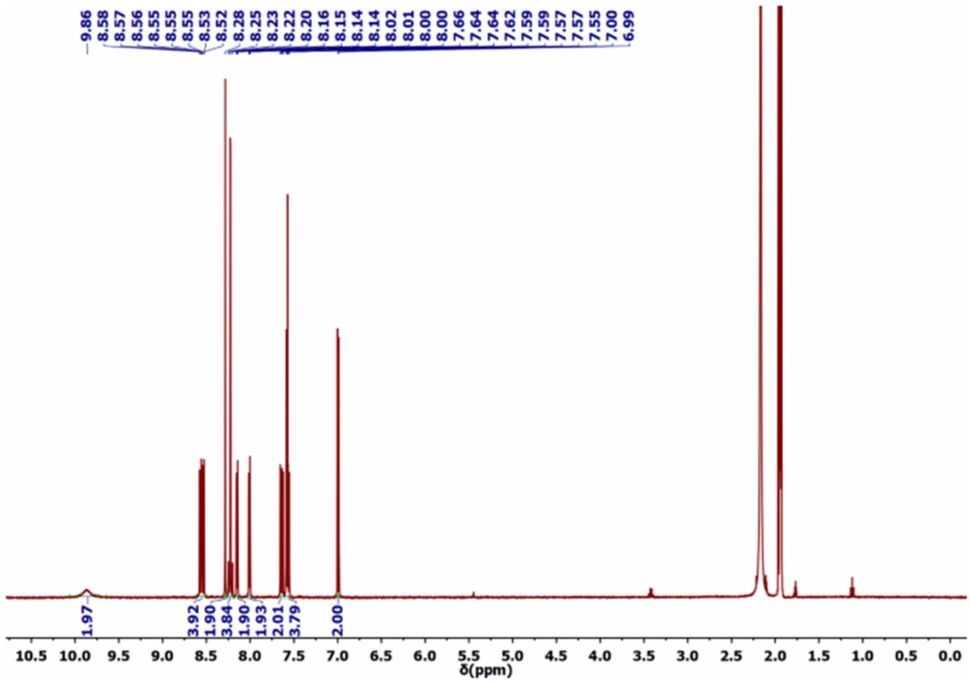

Fig. S1. ^{1}H NMR spectrum of RuPo in CD_3CN.

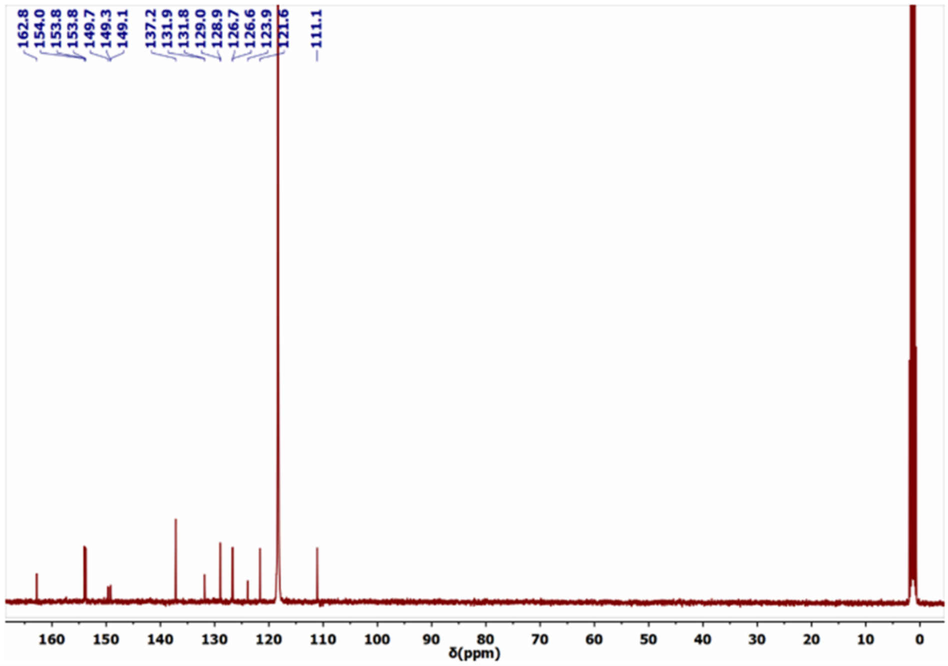

Fig. S2. ^{13}C NMR spectrum of RuPo in CD_3CN.

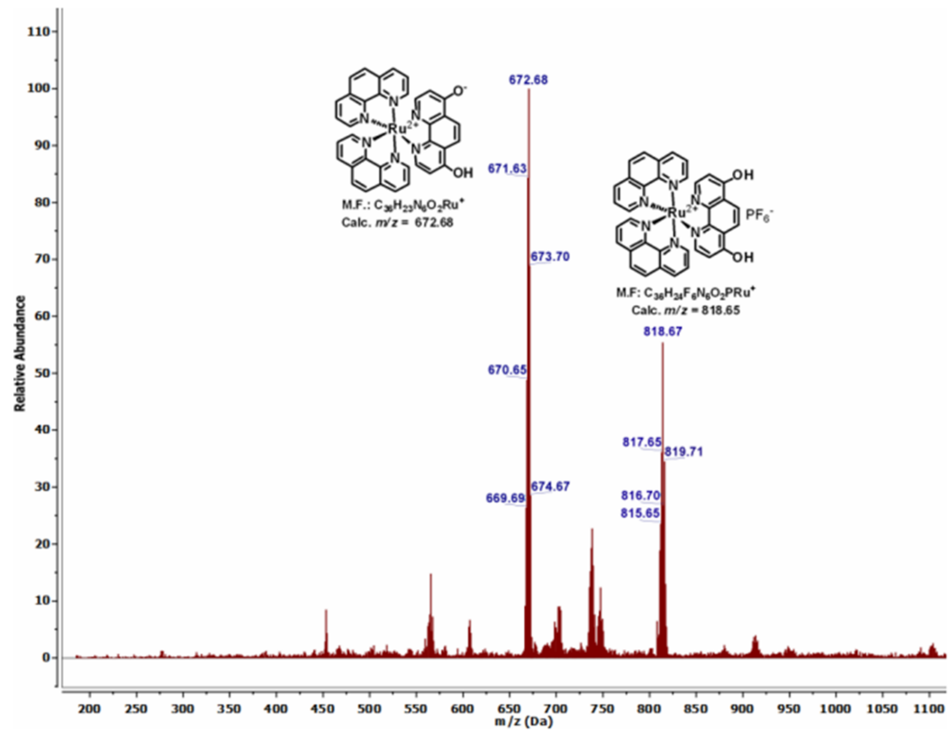

Fig. S3. ESI-MS spectrum of RuPo in CH_3CN.

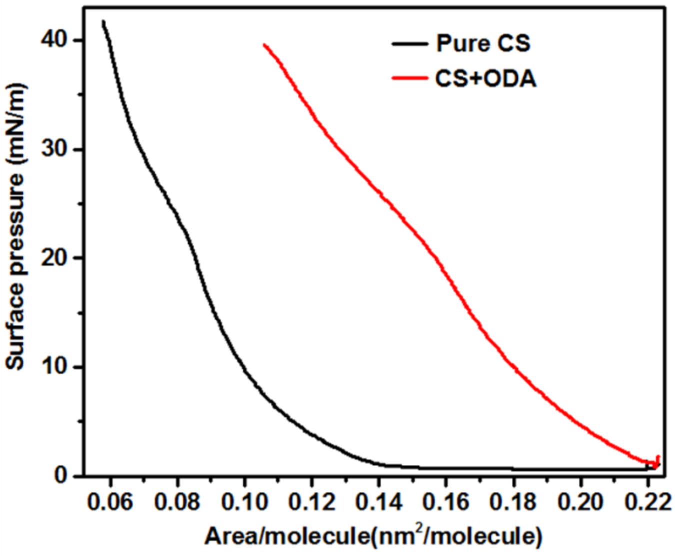

Fig. S4. Isotherm of pure CS and CS+ODA.

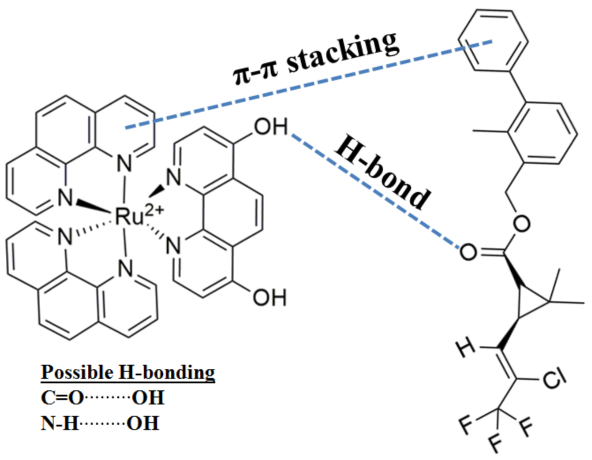

Fig. S5. Schemantic mechanism for interaction of the RuPo with the BF pesticide.

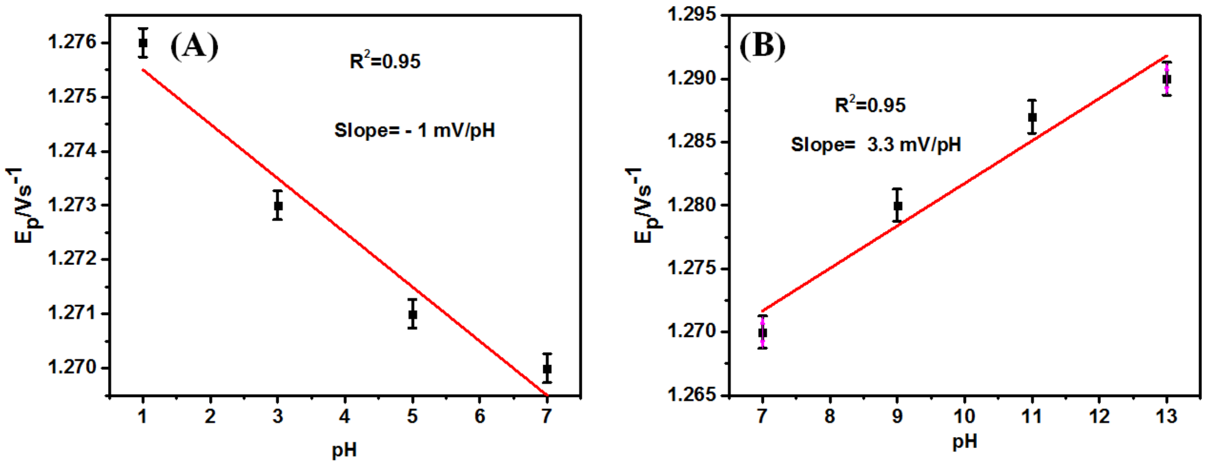

Fig. S6. (A) Plot of E_p(V/s) versus pH (1-7), (B) Plot of E_p(V/s) versus pH (7-13).

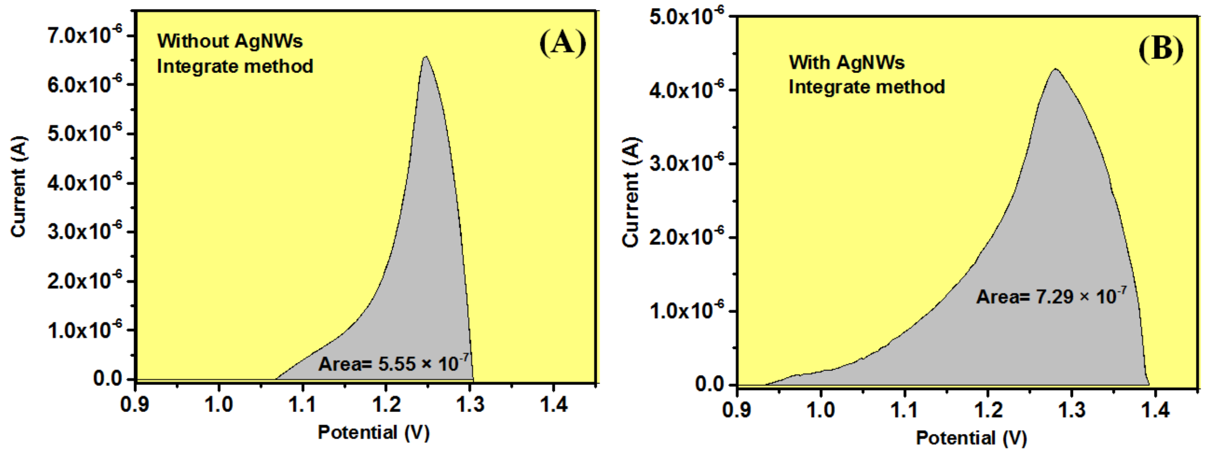

Fig. S7. Area of the DPV curve for (A) without AgNWs, (B) with AgNWs.

.

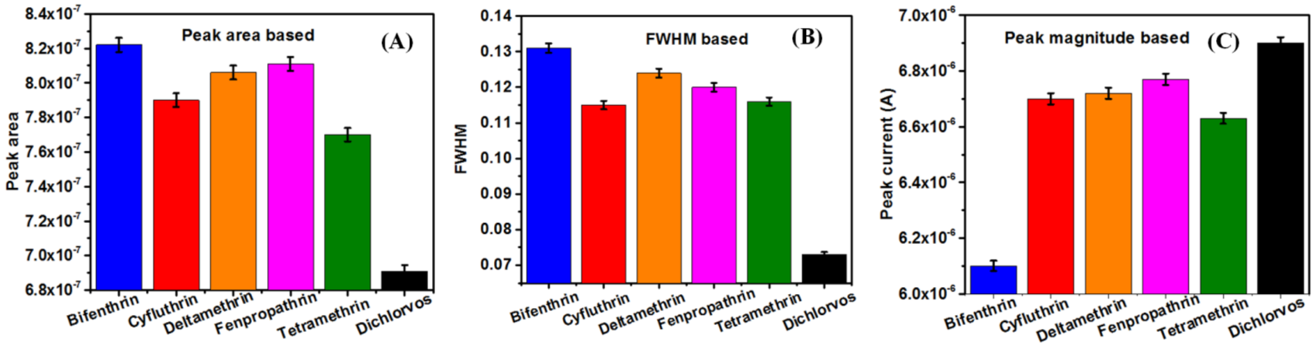

Fig. S8. Bar plot of difereent pesticide versus (A) peak area based, (B) FWHM based, (C) peak magnitude based.

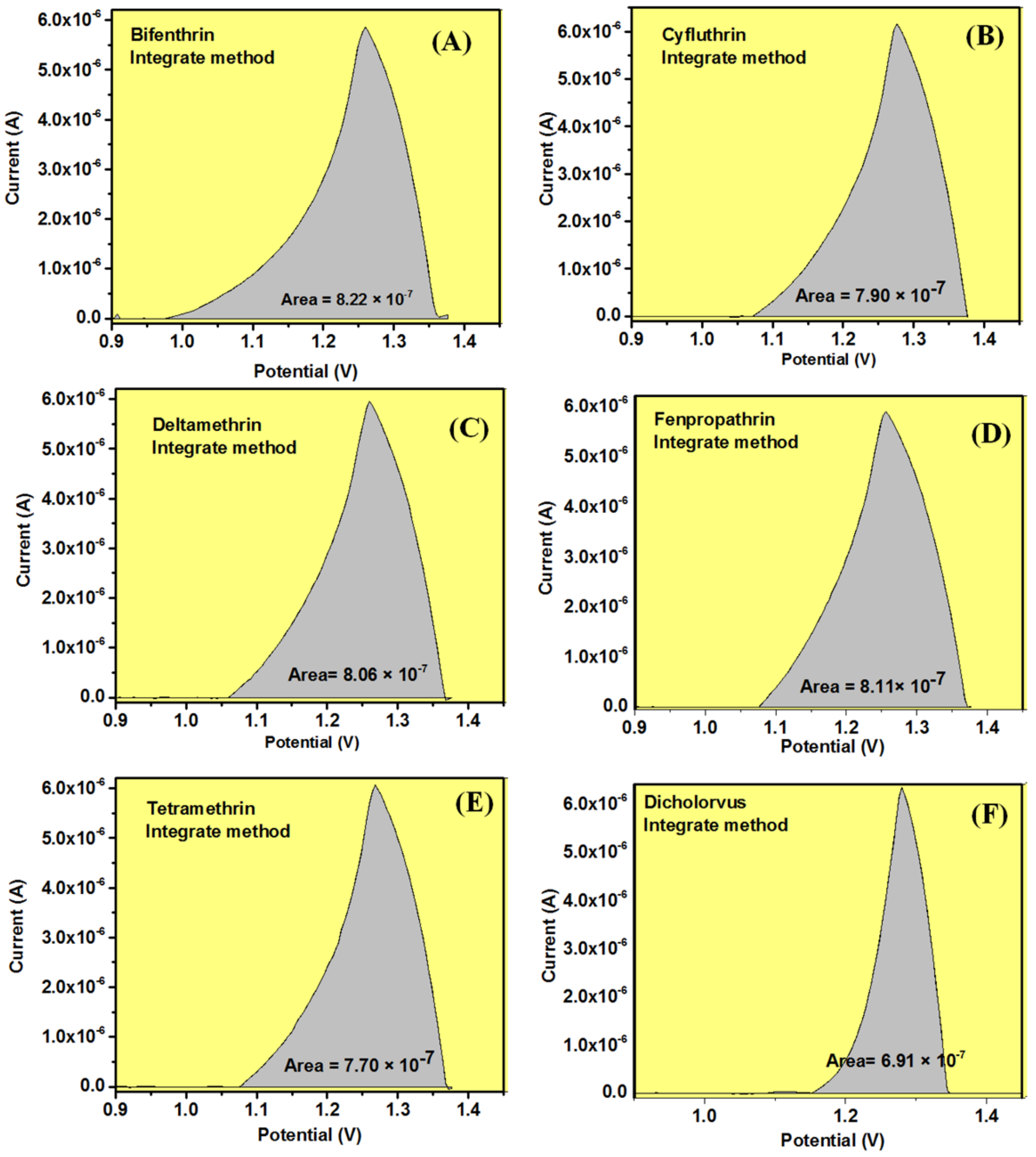

Fig. S9. Peak area of the DPV curve for different pesticides (A-F).

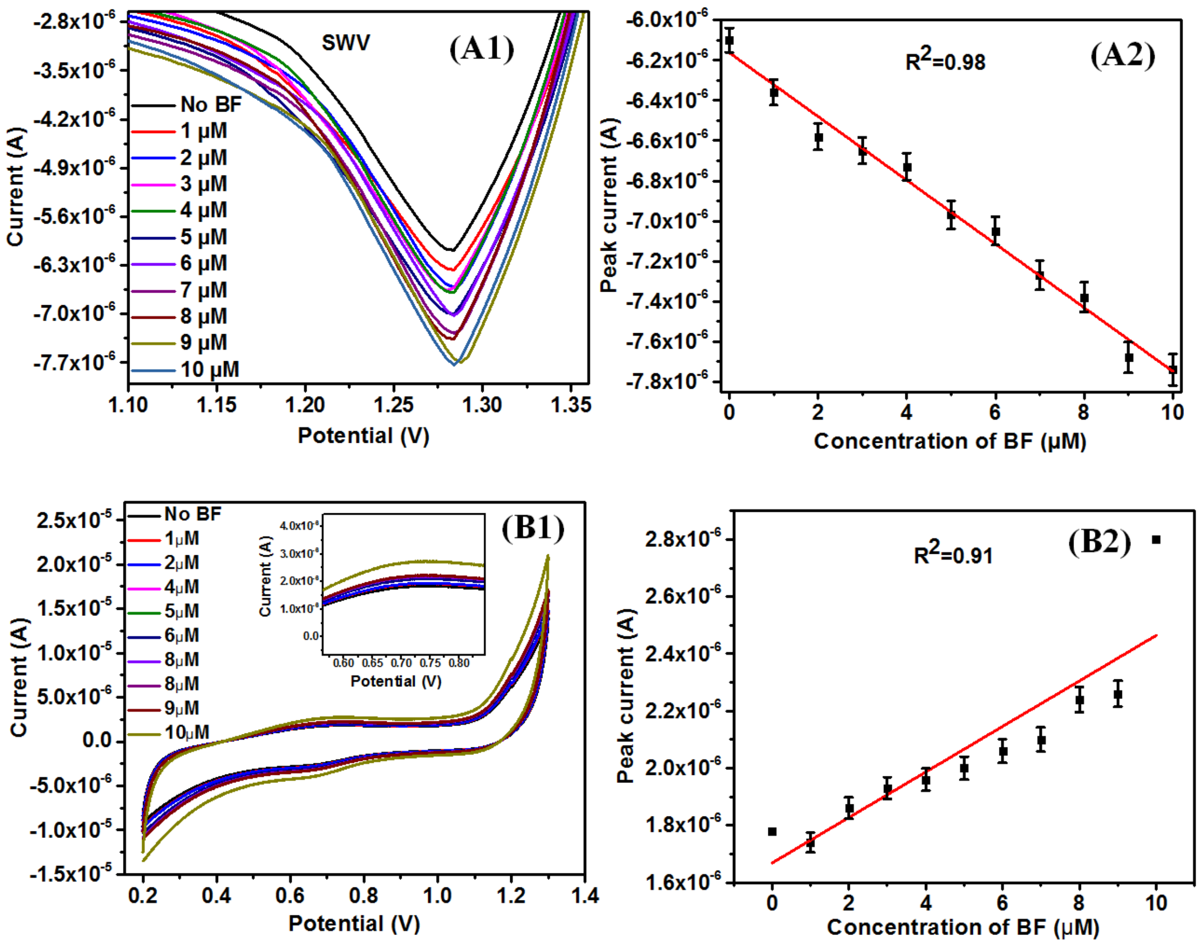

Fig. S10. (A1) SWV graph of composite modified Pt electrode with different concentration of bifenthrin, (A2) Calibration curve with analyte (bifenthrin pesticide) concentrations vs peak current (B1) CV graph of composite modified Pt electrode with different concentration of bifenthrin, (B2) Calibration curve with analyte (bifenthrin pesticide) concentrations vs peak current

Table S1: Table for FTIR peak assignment of the components and composite LB film.

S. No	Peak	Functional group identification	References
1	843	P-F stretching of RuPo	[41]
2	1426	P-F stretching of RuPo	[41]
3	1523	N-H stretching of chitosan	[54]

4	3360	O-H stretching of chitosan	[89]
5	3370	N-H stretching of pure ODA	[90]
6	3373	N-H stretching of ODA in composite	[90]
7	3380	N-H stretching of ODA	[88]
8	3386	N-H stretching of RuPo	[91]
9	3443	N-H stretching of RuPo	[42]
10	3742	–OH group of chitosan	[91]

.

****
